# Supplementary material for: Mobility Assessment Using Multi-Positional MRI in Children with Cranio-Vertebral Junction Anomalies
Source: J Clin Med. 2023 Oct 24;12(21):6714. doi: 10.3390/jcm12216714 (PMC10650482; doi:10.3390/jcm12216714)
Supplement: Supplementary file 1 [file jcm-12-06714-s001.zip › jcm-2649101-supplementary.docx]

**Table S1**. Logic regression for prediction of hypermobility in static neutral position

| Variables | *p-value* |
| --- | --- |
| Age | *0.1* |
| pB-C2 | *0.9* |
| Klaus index | *0.5* |
| C2 retroversion | *0.6* |
| Boogaard angle | *0.8* |
| Platybasia | *0.9* |
| Tentorial angle | *0.3* |
| CXA | *0.3* |
| BDI | *0.8* |
| BAI | *0.1* |

Significant if p-values lower than 0.05; CXA: clivo-axial angle;

BDI: basion-dens interval; BAI: basion-axis interval
